# Serous Adenoma of the Pancreas With Multiple Microcysts Communicating With the Pancreatic Duct

**DOI:** 10.1155/1998/29353

**Published:** 1998-08

**Authors:** S. Samel, F. Horst, H. Becker, U. Brinck, H. Schwörer, G. Ramadori, J.-W. Oestmann

**Affiliations:** 1 Dept. of Surgery Georg-August-University Göttingen Germany; 2 Dept. of Pathology Georg-August-University Göttingen Germany; 3 Dept. of Medicine Georg-August-University Göttingen Germany; 4 Dept. of Radiology Georg-August-University Göttingen Germany; 5 Dept. of Surgery Georg-August-University Göttingen Robert-Koch-Str. 40 Göttingen D-37073 Germany

## Abstract

The rare neoplastic cystic adenomas of the pancreas form two groups of tumors: macrocystic mucinous and microcystic serous adenomas. Both entities show specific radiologic and histologic features. Several recent case reports, however, suggest some diversity within the group of microcystic serous adenomas. We present the case of a young man operated because of epigastric pain for 12 months and a palpable microcystic tumor of the pancreatic head. Multiple cysts communicating with branches of the pancreatic duct in an alveolar-like pattern were demonstrated on endoscopic retrograde cholangiopancreatography. Histologic examination of the specimen confirmed the diagnosis of a serous adenoma of the pancreas. The tumor morphology in this case may suggest a ductal origin of microcystic serous adenomas.


*HPB Surgery*, 1998, Vol. 11, pp. 43–49
Reprints available directly from the publisher
Photocopying permitted by license only

© 1998 OPA (Overseas Publishers Association) N.V.
Published by license under
the Harwood Academic Publishers imprint,
part of The Gordon and Breach Publishing Group.
Printed in India.

### *Case Report and Review of Literature*

### Serous Adenoma of the Pancreas with Multiple Microcysts Communicating with the Pancreatic Duct

S. SAMEL[a,*], F. HORST[a], H. BECKER[a], U. BRINCK[b], H. SCHWÖRER[c], G. RAMADORI[c] and J.-W. OESTMANN[d]

[a] *Dept. of Surgery,* [b] *Dept. of Pathology,* [c] *Dept. of Medicine,*
[d] *Dept. of Radiology, Georg-August-University, Göttingen, Germany*

*(Received 20 June 1997)*

**The rare neoplastic cystic adenomas of the pancreas form two groups of tumors: macrocystic mucinous and microcystic serous adenomas. Both entities show specific radiologic and histologic features. Several recent case reports, however, suggest some diversity within the group of microcystic serous adenomas. We present the case of a young man operated because of epigastric pain for 12 months and a palpable microcystic tumor of the pancreatic head. Multiple cysts communicating with branches of the pancreatic duct in an alveolar-like pattern were demonstrated on endoscopic retrograde cholangiopancreatography. Histologic examination of the specimen confirmed the diagnosis of a serous adenoma of the pancreas. The tumor morphology in this case may suggest a ductal origin of microcystic serous adenomas.**

*Keywords:* Serous cystadenoma, microcystic adenoma, pancreas, pathology, imaging, CT, US, ERCP

### INTRODUCTION

In 1962 Campbell and Cruickshank [2] proposed to distinguish cystic pancreatic tumors with large cysts containing mucous from microcystic tumors with no evident mucous. This classification was reconfirmed again by Compagno and Oertel in 1978 [3] on a series of 34 cases: (1) a group of tumors with a single or multiple macrocysts (>20 mm) lined by mucin-producing cells called mucinous cystadenoma or macrocystic adenoma (MCA) and (2) a microcystic type with serous, glycogen-rich cytoplasm called serous cystadenoma or microcystic adenoma (SCA). Whereas the group of MCAs contains both cystadenoma and cystadenocarcinoma,

*Address for correspondence: Dept. of Surgery, Georg-August-University Göttingen, Robert-Koch-Str. 40, D-37073 Göttingen Germany.

malignant transformations of serous microcystic adenomas have been reported only twice so far [8, 20]. Serous cystadenoma usually occurs in the middle-aged woman with a history of epigastric pain and a palpable epigastric mass in a quarter of cases. It is frequently associated with diabetes mellitus, cholecystolithiasis and benign or malignant, extra- or intra-pancreatic tumor growth [13].

Both entities show specific radiologic and histologic features. The tumor and cyst size may be determined by either ultrasound (US) or computed tomography (CT). On endoscopic retrograde cholangiopancreatography the main pancreatic duct (MPD) usually appears normal or compressed by a SCA. A communication between cysts and the MPD is suggestive of MCA or other cystic lesions of the pancreas. Over the years several case reports, however, suggest some morphologic diversity within the group of serous cystadenomas. Lewandrowski *et al.* [15] and Mori *et al.* [16] describe a serous cystadenoma of the pancreas bearing cysts larger than 20 mm. Egawa *et al.* [4] observed serous ill-defined tumors of the pancreatic head extending into the pancreatic parenchyma which consist of only few microcysts. The communication of a cystic tumor cavity with the ductal system of the pancreas as recently been reported by Furukawa *et al.* [6].

We report a case of SCA where multiple microcysts communicated with branches of the main pancreatic duct. The unusual tumor morphology in this case may again raise the question of the cellular origin of SCA.

### CASE REPORT

A 36-year-old Turkish man was admitted because of a palpable epigastric mass and a history of sporadic epigastric pain for 12 month. Physical examination and upper intestinal endoscopy had repeatedly been without pathological findings and blood chemical values had been normal several times until finally an abdominal CT scan revealed a inhomogenic tumor of the pancreatic head 1 week prior to admission.

On admission a large tender epigastric mass was palpated. Physical examination was otherwise normal. The patient denied nausea or vomiting, recent weight loss, jaundice, dark urine, changes in bowel habits, former pancreatitis or any other major diseases and previous abdominal operations. Neither cholecystolithiasis, diabetes mellitus, pancreatitis nor other associated diseases were diagnosed during examinations.

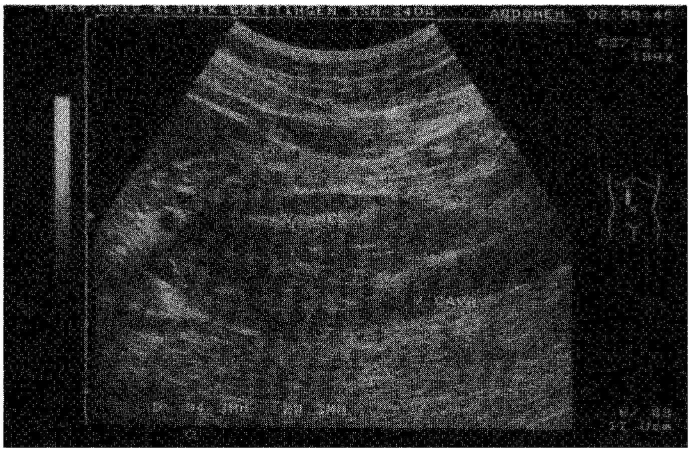

FIGURE 1 Ultrasound demonstrates a well demarcated inhomogeneous enlargement of the pancreatic with multiple hypoechoic areas.

US showed an oval mass arising from the pancreatic head with multiple small echofree areas lined by echogenic material (Fig. 1). Body and tail of the pancreas showed a normal echogenic patter. The main pancreatic duct and the biliary system were normal without signs of compression or occlusion.

CT of the abdomen obtained 1 week prior to admission showed a large inhomogeneous mass arising from the pancreatic head with a large apical radiolucent area (Fig. 2).

ERCP demonstrated multiple cysts of various size communicating with the main pancreatic duct (Fig. 3). The MPD showed a normal morphology.

On laparotomy a well demarcated multicystic enlargement of the pancreatic head adjacent to the duodenum but without signs of invasive

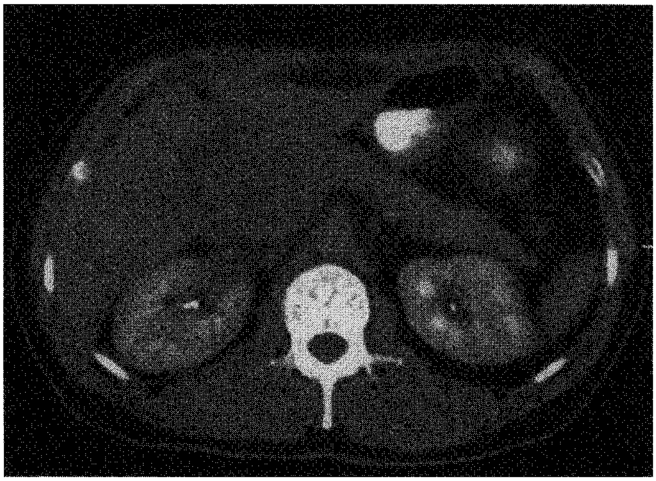

FIGURE 2 CT Scan of the upper abdomen showing a inhomogeneous enlargement of the pancreatic head with a large apical radiolucent area.

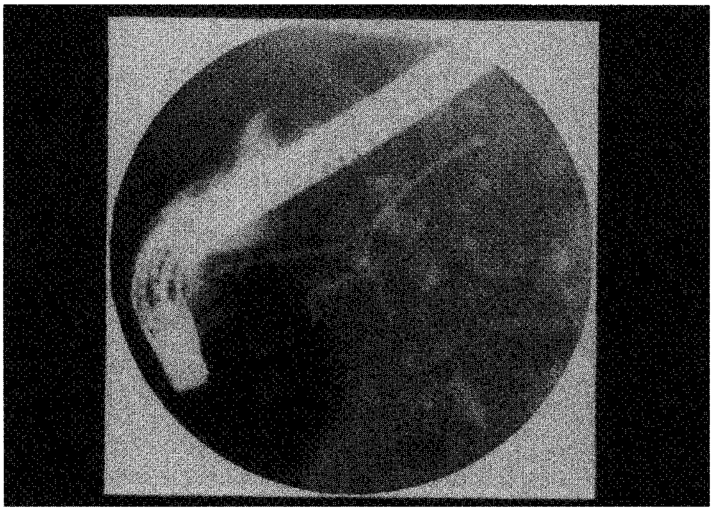

FIGURE 3 Radiograph from an ERCP series documents multiple cystic communications with the pancreatic duct system.

FIGURE 4 An Intraoperative macroscopic photograph demonstrates a well defined cystic tumor arising from the pancreatic head adjacent to the duodenal convexity. (See Color Plate I).

growth was found (Fig. 4). A Traverso procedure (duodenum-preserving pancreatic head resection) was performed.

Specimen radiographs of the excised tumor after cannulation of the MPD showed fine calcifications within the tumor and a single calcified cystic structure. After injection of contrast material multiple cysts were filled via communications with otherwise normally configured ductal branches (Fig. 5).

The specimen (Fig. 6) was sent for histologic examination. The patient recovered well and

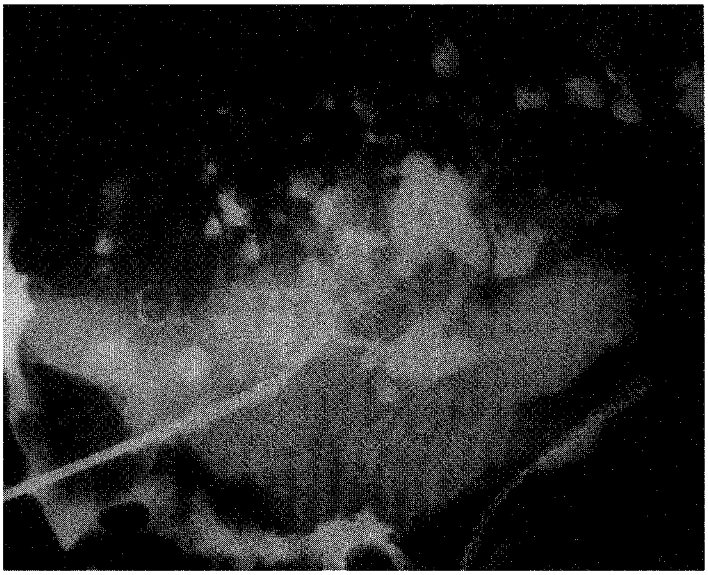

FIGURE 5 A radiograph of the specimen after injection of contrast media into the ductal system reveals multiple cysts communicating with ductal branches. A shell-like calcification and multiple parenchymal calcifications are also demonstrated.

FIGURE 6 Gross macroscopic photograph of the resected multilocular cystic tumor. (See Color Plate II).

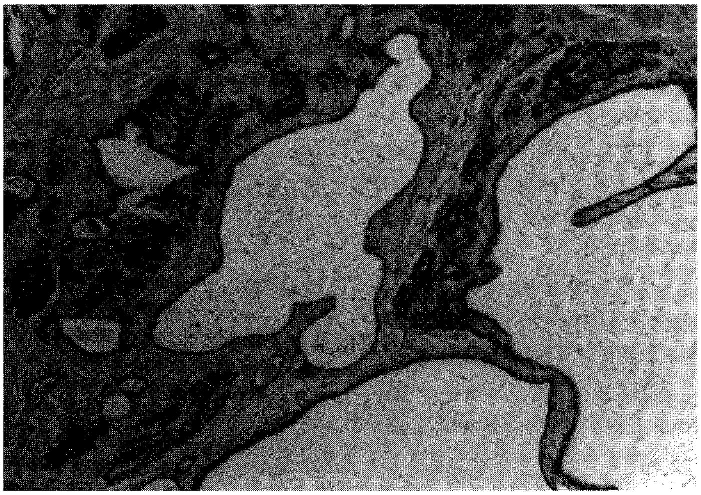

FIGURE 7 Photomicrograph of the tumor showing small islets of pancreatic parenchyma among large cyst of various diameter containing a serous liquid lined by a single layer of isoprismatic cells.

was discharged from hospital 10 days after surgery.

### Pathologic Findings

Macroscopically, the ovoid and well circumscribed tumor arising from the pancreatic head measuring 95x80x50 mm consisted of multiple cysts of various sizes. The tumor surface was smooth, well defined and free of solid nodules (Fig. 5). The pancreatic parenchyma appeared to be of normal consistency and the proximal pancreatic duct was of regular shape.

Microscopically, cross sections of the tumor showed cysts of various size but smaller than 20 mm containing an eosinophilic liquid. The cysts were lined by a single layer of glycogenrich flat or cuboidal cells with small pleomorphic nuclei (Fig. 6). Chromogranin- and Ki67-labelling indices were 1%. No mucous nor mucinous or myoepithelial cells were found. The cysts appeared to be trapped in a webwork of partially fibrotic pancreatic parenchyma with few islets of normal pancreatic tissue, regular as well as degenerated acinar structures and small intralobular ducts.

### DISCUSSION

Large case series published since the 1970s have contributed to the present understanding of the neoplastic cystic tumors of the pancreas [3, 9, 19, 1, 18; 5, 12, 21]. Single case reports have added rare aberrant features e.g., a macro-cystic [15, 16] or oligocystic [4] structure, communication between cysts and the pancreatic ductal system [6], multifocal tumororigin [14], associated tumor-growth [13] and malignant transformation [8, 20].

Serous cystadenomas usually arise from the head or body of the pancreas and seldom involve the entire organ. Their typical sonographic and radiologic feature is a uniform composition of multiple small (<2 cm) cysts. They show a central fibrous scar and "sunburst" calcifications throughout the tumor stroma and sometimes signs of hypervascularisation [10, 11]. On ERCP the MPD is either normal, displaced or compressed by the tumor mass [7]. Their macroscopic aspect is that of an ovoid well defined tumor with a smooth but irregular shape due to their polycystic composition. Frequently there is fibrous sometimes calcified tissue trapped in-between the cysts. Their microscopic pattern is characterized by the uniform architecture of the cysts lined by flat or cuboidal glycogen-rich epithelium [3]. The ultrastructure of these epithelial cells displays traits of ductal, centroacinar, acinar and islet cell of which the centroacinar cell seems to share the most similarities with the neoplastic epithelium [1].

The common detection of intracytoplasmic glycogen and the occasional finding of myoepithelial cells in SCA [17] has raised the question whether the origin of SCA may be the more proximal ductal cell. In the human pancreas myoepithelium can be found only in the MPD.

The case of SCA we report above displays most of the common macro- and microscopic characteristics of such tumors, yet it presents another incidence of serous cystadenoma of the pancreas where cysts communicate with the pancreatic duct. The described tumor, however, differs from the lesion seen by Furukawa *et al.*, where a single cyst communicated with the MPD. In our case ERCP demonstrated various cysts in communication with the branches of the MPD in an alveolar-like pattern. It seems to be unlikely that such a pattern of communication is due to erosion of the pancreatic duct by growing cysts as it has been hypothesized by Furukawa [6]. Our findings rather suggest that SCA may arise from ductal epithelium proximal of the centroacinar cell. Positive Chromagranin-staining of cystic epithelium as found in our specimen is indicative of ductal cells [1]. However, we did not find any myoepithelial cells in the tumor that would further support the hypothesis of a ductal origin of these tumors. Careful examination of further cases of serous cystadenoma is needed to further clarify the histogenesis of this rare pathological entity.

### *References*

[1] Alpert, L. C., Truong, L. D., Bossart, M. I. and Spjut, H. J. (1988). Microcystic adenoma (serous cystadenoma of the Pancreas. *A.J.S.P.*, **12**, 251–263.

[2] Campbell, J. A. and Cruickshank, A. H. (1962). Cystadenoma and cystadenocarcinoma of the pancreas. *J. Clin. Pathol.*, **15**, 432–437.

[3] Compagno, J. and Oertel, J. E. (1978). Microcystic adenomas of the pancreas (Glycogen-rich cystadenomas). A clinico-pathologic study. *Am. J. Clin. Pathol.*, **69**, 289–298.

[4] Egawa, N., Maillet, B., Schröder, S., Mukai, K. and Klöppel, G. (1994). Serous oligocystic and ill-demarcated adenoma of the pancreas: A variant of serous cystic adenoma. *Virchows Archiv.*, **424**, 13–17.

[5] Fabiani, A., Delia, G. J., De Rosa, R. *et al.* (1996). Pancreatic serous cystadenomas. Report of 8 cases with a mean follow up of 7 years. *HPB Surg.*, **9**, 215–217.

[6] Furukawa, H., Takayasu, K., Mukai, K. *et al.* (1996). Serous cystadenoma of the pancreas communicating with a pancreatic duct. *Int. J. Pancreatol.*, **19**, 141–144.

[7] Gazelle, G. S., Müller, P. R., Raafat, N., Halpern, E. F., Cardenosa, G. and Warshaw, A. L. (1993). Cystic neoplasms of the pancreas: Evaluation with endoscopic retrograde pancreatography. *Radiology*, **188**, 633–636.

[8] George, D. H., Murphy, F., Michalski, R. and Ulmer, B. G. (1989). Serous cystadenocarcinoma of the pancreas: A new entity? *Am. J. Surg. Pathol.*, **13**, 61–66.

[9] Hodgkinson, D. J., Remine, W. H. and Weiland, L. H. (1978). Pancreatic cystadenoma. *Arch. Surg.*, **113**, 512–519.

[10] Itai, Y., Ohhashi, K., Furui, S., Araki, T., Murakami, Y., Ohtomo, K. and Atomi, Y. (1988). Microcystic adenoma of the pancreas: Spectrum of computed tomographic findings. *J. Comput. Assist. Tomogr.*, **12**, 797–803.

[11] Johnson, C. D., Stephens, D. H., Chaboneau, J. W., Carpenter, H. A. and Welsh, T. J. (1988). Cystic pancreatic tumors: CT and sonographic assessment. *A.J.R.*, **151**, 1133–1138.

[12] Kamei, K., Funabiki, T., Ochiai, M., Amano, H., Marugami, Y., Kasahara, M. and Sakamato, T. (1992). Some considerations on the biology of pancreatic serous cystadenoma. *Int. J. Pancrea.*, **11**, 97–104.

[13] Keel, S. B., Zukerberg, L., Graeme Cook, F. and Compton, C. C. (1996). A pancreatic endocrine tumor arising within a serous cystadenoma of the pancreas. *Am. J. Surg. Pathol.*, **20**, 471–475.

[14] Kim, Y. I., Seo, J. W., Suh, J. S., Lee, K. U. and Choe, K. J. (1990). Microcystic Adenomas of the Pancreas. *A.J.C.P.*, **94**, 150–156.

[15] Lewandrowski, K., Warshaw, A. and Compton, C. (1992). Macrocystic Serous cystadenoma of the pancreas. *Human Pathol.*, **23**, 871–875.

[16] Mori, K., Takeyama, S., Hirosawa, H. *et al.* (1995). A case of macrocystic serous cystadenoma of the pancreas. *Int. J. Pancreatol.*, **17**, 91–93.

[17] Nyongo, A. and Huntrakoon, M. (1985). Microcystic adenoma of the pancreas with myoepithelial cells. *A.J.C.P.*, **84**, 114–120.

[18] Pyke, C. M., van Heerden, J. A., Colby, T. V., Sarr, M. G. and Weaver, A. L. (1992). The spectrum of serous cystadenoma of the pancreas. *Ann. Surg.*, **215**, 132–139.

[19] Shorten, S. D., Hart, W. R. and Petras, R. E. (1986). Microcystic adenomas (serous cystadenomas) of the pancreas. *A.J.S.P.*, **10**, 365–72.

[20] Yoshimi, N., Sugie, S., Tanaka, T., Aijin, W., Bunai, Y., Tatematsu, A., Okada, T. and Mori, H. (1992). A rare case of serous cystadenoma of the pancreas. *Cancer*, **69**, 2449–2453.

[21] Zanow, J., Gellert, K., Benhidjeb, T. and Muller, J. M. (1996). [Cystic tumors of the pancreas] Cystische tumoren des pankreas. *Chirurg.*, **67**, 719–724.

